# Pharmacological and cell-based treatments to increase local skin flap viability in animal models

**DOI:** 10.1186/s12967-024-04882-9

**Published:** 2024-01-17

**Authors:** Charlotte E. Berry, Thalia Le, Nicholas An, Michelle Griffin, Micheal Januszyk, Carter B. Kendig, Alexander Z. Fazilat, Andrew A. Churukian, Phoebe M. Pan, Derrick C. Wan

**Affiliations:** Hagey Laboratory for Pediatric Regenerative Medicine, Division of Plastic and Reconstructive Surgery, Department of Surgery, Stanford University School of Medicine, 257 Campus Drive West, Stanford, CA 94305 USA

**Keywords:** Skin flap, Cell-based therapy, Flap viability, Pharmacologic treatment

## Abstract

Local skin flaps are frequently employed for wound closure to address surgical, traumatic, congenital, or oncologic defects. (1) Despite their clinical utility, skin flaps may fail due to inadequate perfusion, ischemia/reperfusion injury (IRI), excessive cell death, and associated inflammatory response. (2) All of these factors contribute to skin flap necrosis in 10–15% of cases and represent a significant surgical challenge. (3, 4) Once flap necrosis occurs, it may require additional surgeries to remove the entire flap or repair the damage and secondary treatments for infection and disfiguration, which can be costly and painful. (5) In addition to employing appropriate surgical techniques and identifying healthy, well-vascularized tissue to mitigate the occurrence of these complications, there is growing interest in exploring cell-based and pharmacologic augmentation options. (6) These agents typically focus on preventing thrombosis and increasing vasodilation and angiogenesis while reducing inflammation and oxidative stress. Agents that modulate cell death pathways such as apoptosis and autophagy have also been investigated. (7) Implementation of drugs and cell lines with potentially beneficial properties have been proposed through various delivery techniques including systemic treatment, direct wound bed or flap injection, and topical application. This review summarizes pharmacologic- and cell-based interventions to augment skin flap viability in animal models, and discusses both translatability challenges facing these therapies and future directions in the field of skin flap augmentation.

## Introduction

**Fig. 1:**
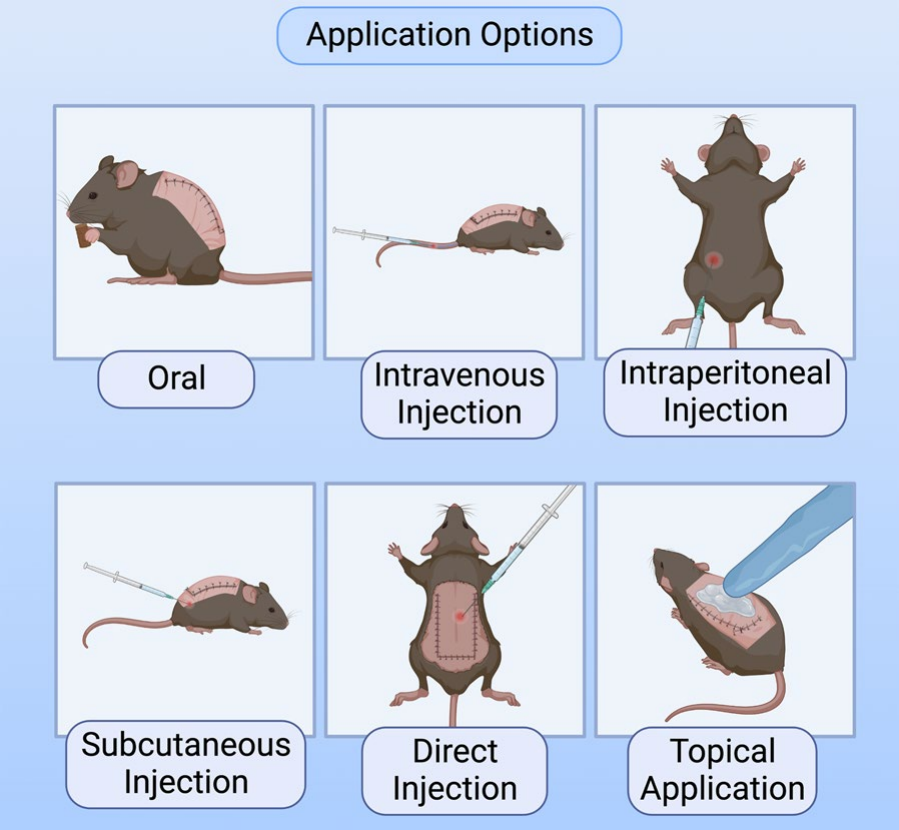
Therapeutics can be applied to skin flaps through various modalities. Six common modalities are visualized: oral, intravenous injection, intraperitoneal injection, subcutaneous injection, direct injection, and topical application. These are demonstrated on a murine McFarlane skin flap model

Local skin flaps are frequently employed for wound closure to address surgical, traumatic, congenital, or oncologic defects [1]. Despite their clinical utility, skin flaps may fail due to inadequate perfusion, ischemia/reperfusion injury (IRI), excessive cell death, and associated inflammatory response [2]. All of these factors contribute to skin flap necrosis in 10–15% of cases and represent a significant surgical challenge [3, 4]. Once flap necrosis occurs, it may require additional surgeries to remove the entire flap or repair the damage and secondary treatments for infection and disfiguration, which can be costly and painful [5]. In addition to employing appropriate surgical techniques and identifying healthy, well-vascularized tissue to mitigate the occurrence of these complications, there is growing interest in exploring cell-based and pharmacologic augmentation options [6]. These agents typically focus on preventing thrombosis and increasing vasodilation and angiogenesis while reducing inflammation and oxidative stress. Agents that modulate cell death pathways such as apoptosis and autophagy have also been investigated [7]. Implementation of drugs and cell lines with potentially beneficial properties have been proposed through various delivery techniques including systemic treatment, direct wound bed or flap injection, and topical application (Fig. 1).

This review summarizes pharmacologic- and cell-based interventions to augment skin flap viability in animal models, and discusses both translatability challenges facing these therapies and future directions in the field of skin flap augmentation.

### Pharmacologic treatments

First-line interventions for improving flap viability typically include surgical technique optimization and hemodynamic support, usually followed by pharmacological or cell-based therapies as second-line interventions to ensure the best flap survival. Insufficient blood supply and IRI are the two main causes of distal flap necrosis [2]. To improve blood supply and decrease the incidence and effects of IRI, a variety of pharmacologic interventions have been explored for their observed or theoretical utility in improving skin flap viability. While many medications have several benefits, they will be discussed broadly as antithrombotic agents, vasodilators, pro-angiogenic therapies, antioxidants and anti-apoptotics, upregulators of autophagy, and anti-inflammatory drugs.

#### Antithrombotic agents

Arterial or venous thrombosis can impair capillary circulation and nutrient supply to the flaps [2, 8]. Venous obstruction occurs more frequently than arterial obstruction due to lower flow rate and thin, friable nature of veins. Thrombosis could be induced via two mechanisms: (1) increased platelet and neutrophil adhesivity due to local injury that releases free radicals, enzymes, and cytokines to obstruct the capillaries and (2) activation of the coagulation cascade to form a clot [8]. Anti-thrombotic drugs can reduce thrombotic risk and improve blood circulation to reduce tissue necrosis and improve flap viability (Table 1). They can be categorized into two groups: anti-platelet agents or antithrombin activator/clotting factor inhibitors. Clinically, both aspirin and heparin are given empirically to patients prior to undergoing skin flap surgery with the goal of improving circulation and thereby flap survival.

**Table 1:** Anti-thrombotic therapies

Pharmacologic therapy	MoA	RoA	Animal model	Treatment protocol	References
Low-molecular-weight heparin	Enhancement of Antithrombin III	Subcutaneous injection	Rabbit congested pedicled flaps	Single immediate post-operative injection	Miyawaki et al. [9]
Hirudin	Enhancement of Antithrombin III (contains the active ingredient heparinoid)	Subcutaneous injection	Rat caudally-based random dorsal flap	Immediate injection after surgery and again on postoperative days 3 and 5, 1–3 cm proximal to the distal limit of the flap	Yingxin et al. [10]
Hirudoid	Activation of anti-thrombin III (contains the active ingredient heparinoid), Inhibition of hyaluronidase, Stimulation of growth factor production such as VEGF and PDGF	Topical cream	Rat caudally based dorsal flaps	Post-operative application for 9 days	Livaoğlu et al. [8]
Bivalirudin	Direct inhibition of thrombin, enhancement of anticoagulation	Intraperitoneal injection	Rat random dorsal flaps	Post-operative injection once a day for 7 days	Cai et al. [11]
Activated protein C	Inactivation of serine protease clotting factor Va, VIIIa, Xa, and tissue factor. Inhibition of fibrinolysis	Intravenous injection	Rat cranially-based dorsal cutaneous flap	Injections in 3 groups: postoperative, late preoperative, and early preoperative	Bezuhly et al. [12]
Platelet-rich plasma gel	Concentrated form of platelets, growth factors PDGF, TGF-β, platelet-derived epidermal growth factor, platelet-derived angiogenesis factor insulin-like growth factor-1, and platelet factor-4, stimulate the mesenchymal cells and epithelial cells proliferation, and increase collagen and matrix synthesis, fibrinogen, fibronectin, and vinculin	Gel application on the wound	Rats cranially-based dorsal random flap	PRP gel was applied on the wound surface and then the flap was sutured	Chai et al. [13]
		Subcutaneous injection	Rabbit cranially-based dorsal random-pattern flap	Post-operative injection once	Wang et al. [14]

Aspirin is an irreversible inhibitor of the enzyme cyclooxygenase, the key enzyme in metabolizing arachidonic acid. Aspirin has been shown to be highly effective in improving skin flap viability, hence its frequent use in clinical settings. While some studies indicate that this effect is mediated by inflammation modulation and improvement of flap circulation through direct vasodilation more so than an anti-platelet aggregation effect [15], others indicate all three of these effects contribute meaningfully to aspirin’s benefits [16]. Clopidogrel (an irreversible platelet aggregation inhibitor), alternatively, has also been shown to improve skin flap viability but through a primarily antiaggregatory effect [17].

Several anticoagulants have been studied in animal local skin flap survival models, many of which are also employed clinically. Key differences among these agents are their mechanisms of action, routes of administration, and clinical indications. Low molecular weight Heparin (LMWH) and Hirudin are subcutaneously administered anticoagulants, while Bivalirudin and Activated Protein C can be administered intraperitoneally or intravenously. In contrast, Hirudoid is a topical anti-inflammatory, antithrombotic, and fibrinolytic drug with reduced systemic effects. A study in 2019 by Livaoğlu et al. showed that daily topical Hirudoid application on random dorsal skin flaps in rats resulted in significantly lower inflammation, edema, and intravascular thrombosis scores, as well as a smaller flap necrosis area (31.7% vs. 48.9%) compared to control animals [8]. Subcutaneous injection of natural and recombinant Hirudin and intraperitoneal injection of Bivalirudin following creation of random flaps in rats also both increased flap survival rates. The mechanisms underlying this effect may involve reducing thrombosis, improving flap blood supply, and upregulation of vascular endothelial growth factor (VEGF) activity, which promotes formation of new blood vessels in the flap [11, 10].

Another study investigated subcutaneous use of LMWH—which acts by enhancing antithrombin III—in four pedicled-flap models in rabbits and found that LMWH had a rapid therapeutic effect on flap circulation and survival length [9]. All of the congested flaps survived when treated with LMWH, while the control group showed necrosis in one-sixth of the flap area [9]. Finally, intravenous administration of Activated Protein C in rats also showed significantly improved flap survival in the experimental group compared to the control (68.9% vs. 39.3%, respectively) 1 week after the injection [12]. Interestingly, potentially due to time-sensitive transcriptional changes, earlier Activated Protein C injections prior to elevation of the flap were associated with higher flap survival [12]. However, disadvantages of Activated Protein C treatment include increased risk for significant hemorrhage during flap dissection when administered preoperatively, short half-life of the drug, and potential need for supraphysiologic doses as much as 1000-fold higher than those safely used in patients [12].

#### Vasodilators

Vasoconstriction following skin flap elevation is a common cause of flap necrosis [18]. Release of norepinephrine from flap dissection and injured sympathetic neurons produces a local hyperadrenergic environment [19]. Consequently, nutrient-rich blood flow to the distal aspect of the flap may become restricted, and blood is redirected through low-pressure arteriovenous shunts, increasing the risk of irreversible ischemic necrosis [18, 19]. Vasodilators to improve flap viability (Table 2) act directly on arteriolar smooth muscle and can be categorized into three groups: those that target the nitric oxide synthase (NOS) pathway to release nitric oxide (NO), those that target the NOS pathway in combination with other mechanisms, and those that induce vasodilation through non-NOS-mediated mechanisms.

**Table 2:** Vasodilatory therapies

Pharmacologic therapy	MoA	RoA	Animal model	Treatment protocol	References
NO donors: sodium nitroprusside (SNP) and diethylenetriamine NONOate (DETA-NO)	NO-mediated vasodilation and angiogenesis	Subcutaneous injection or transdermal ionophoretic delivery	Rat caudally-based dorsal random pattern flap	Post-operative injection for 5 days	Russell et al. [20]
Metformin	AMPK mediated phosphorylation of NO synthase, increasing NO bioavailability and inducing vasodilation	Intraperitoneal injection	Rat caudally-based dorsal random pattern flap	Pre-treatment 4 h pre-operatively	Taleb et al. [18]
PDE-5 inhibitor	Inhibits cGMP degradation in the NO pathway, leading to NO persistance	Intraperitoneal injection	Rat dorsal McFarlane-type flap	Post-operative injection for 7 days, once or twice daily	Shah et al. [21]
Azelaic acid, minoxidil, caffeine	Inhibits DHT synthesis and activates ATP-sensitive potassium channels	Topical	Rat cephalically-based dorsal random pattern flap	Post-operative application for 7 days	Farrokhi et al. [22]
Vasonatrin	Venodilating actions of CNP, natriuretic action of ANP, and the arterial vasodilating actions not associated with either	Intravenous injection	Rat caudally-based dorsal random pattern flap	Post-operative injection once daily for 3 days	Wang et al. [23]
Calcium channel blocker	Arteriolar smooth muscle relaxant	Oral	Rat caudally-based dorsal random pattern flap	(1) Pre-operative administration every 12 h for 1 day and continued post-operatively for 7 days (2) Post-operative every 12 h for 7 days	Pal et al. [24]
Calcium channel blocker	Arteriolar smooth muscle relaxant	Intraperitoneal injection and oral	Rat ventral island flap with random portion	(1) Intraoperative injection followed by oral for 7 days post-op (2) Pre-treatment orally for 5 days, followed by intraoperative injection, followed by oral for 7 days post-operatively	Bailet et al. [19]
Caltonin gene-related peptide	Arterial vasodilation	Intravenous injection	Rat groin island flap	(1) Within 12-h post-operative ischemic period (2) Pre-treatment immediately pre-operatively and within 12-h post-operative ischemic period	Gherardini et al. [25]

Examples of agents that target the NOS pathway include sodium nitroprusside (SNP), diethylenetriamine NONOate (DETA-NO), sildenafil, and metformin. Transdermal iontophoretic delivery of NO donors such as SNP and DETA-NO showed significantly improved perfusion in rat skin flaps [20, 26]. Similarly, pre-operative administration of metformin, a primary therapeutic option for type 2 diabetes, has been shown to enhance NOS activity via the 5′ adenosine monophosphate-activated protein kinase pathway, resulting in significant improvements in vasodilation and flap viability in rats [18].

Vasodilating therapies that target NOS signaling in concert with other pathways include vasonatrin peptide (VNP) and a combination treatment of Azelaic acid, minoxidil, and caffeine (AMC). VNP, a synthetic natriuretic peptide derived from the combination of atrial natriuretic peptide and C-type natriuretic peptide, induces smooth muscle vasodilation. In rats, Wang et al. demonstrated these combined effects resulted in a significant increase in mean vessel diameter, blood perfusion volume, and flap viability while reducing thrombosis, inflammatory mediators, and oxidative injury [23]. Like VNP, studies with AMC on rat skin flaps have shown a significant increase in NOS activity [22]. This effect has been attributed to the 5α-reductase inhibitory properties of azelaic acid—a dermatological treatment for skin conditions—which promotes NOS expression and NO production by inhibiting dihydrotestosterone (DHT) synthesis [22]. Azelaic acid has also been found to enhance levels of the anti-apoptotic protein B-cell lymphoma 2 [22]. Multiple animal studies have underscored the utility of NOS pathway modulators in enhancing local flap perfusion and survival, supported by the ubiquitous reduction in flap necrosis observed [18, 20–22].

Agents that facilitate vasodilation through non-NOS pathways that have been studied to promote flap survival include calcitonin gene-related peptide (CGRP), an endogenous hormone known for its potent vasodilatory effects, and nifedipine, a selective calcium channel blocker commonly prescribed for hypertension [19, 25]. While the precise mechanism of CGRP has not been fully elucidated, some of its effect has been attributed to CGRP receptor binding on smooth muscle cells, while nifedipine's effect is associated with the inhibition of the α-2 receptor. Calcitonin gene-related peptide also exhibits anti-inflammatory properties, while nifedipine possesses antioxidant properties, prevents calcium influx, and inhibits platelet aggregation [19, 25, 24]. These multifaceted therapeutic mechanisms highlight the utility of CGRP and nifedipine in optimizing local skin flap survival in rats [19, 25, 24].

#### Pro-angiogenic therapies

In addition to anti-thrombotic agents and vasodilation to promote blood flow, viability of the distal flap can also be enhanced through vascular formation and remodeling (Table 3) [33]. In ischemic skin flaps, angiogenesis is typically mediated by VEGF and basic fibroblast growth factor (bFGF), which is secreted by keratinocytes and fibroblasts in response to hypoxia [27]. VEGF binds to receptors on the surface of the dermal vascular endothelium to stimulate mitosis, inhibit endothelial cell apoptosis, and enhance vascular permeability and cell migration [27]. Direct injection of VEGF into rat skin flaps has been shown to significantly increase flap viability area (38.9% in the control vs. 80.4% in the VEGF-treated group) [10]. Similarly, upregulation of bFGF expression in the flap bed using plasmid-based methods significantly decreased the area of flap necrosis and enhanced vascularity in dorsal skin flaps. Although both VEGF and bFGF are promising agents to improve vascularization in ischemic flaps, their short half-life, expensive costs, instability requiring complicated storage, and uncertainty regarding effective/safe dosage remain significant limitations which have prompted researchers to investigate alternative angiogenic therapeutics [28, 29].

**Table 3:** Angiogenic therapies

Pharmacologic therapy	MoA	RoA studied	Animal model	Treatment protocol	References
The tetrapeptide acetyl-serine-aspartyl-lysine-proline	Regulation of hematopoiesis peptide	Subdermal injection	Rat dorsal and abdominal skin flaps	Post-operative injections, twice a day for 3 days	Fromes et al. [30]
VEGF	Stimulation of angiogenic growth factors, angiogenesis, vascular permeability. Inhibition of apoptosis in endothelial cells	Subdermal injection	Rat dorsal pedicled skin flaps	Pre-operative injections, once daily, 7 days	Vourtsis et al. [31]
Basic fibroblast growth factor	Stimulation of angiogenic growth factors, angiogenesis, and arteriogenesis	Gene transfer	Rat dorsal axial skin flap	Pre-operative injection of bFGF gene plasmid vector and electroporation 2 days	Fujihara et al. [32]
Epigallocatechin gallate	Enhancement of prostaglandin F2a–induced VEGF synthesis, and protein kinase/c-jun N-terminal kinase	Injection into the interspace between the dermal tissue and subcutaneous membrana carnosa or topical application	Rat dorsal skin flap	+ Local injection five times just before flap elevation + Topical application to the flap just before flap elevation	Cheon et al. [33]
Memantine	An *N*-methyl-*D*-aspartate (NMDA) receptor antagonist used primarily in the treatment of Alzheimer's disease Upregulation of VEGF	Intraperitoneal injection	Rat random skin flap	Daily post-operative injections for 7 days	Fan et al. [2]
Polydeoxyribonucleotide	Upregulation of VEGF	Subdermal or intraperitoneal injection	Rat dorsal skin flap	Subdermal injections distributed evenly in the 12 areas at proximal, middle, and distal areas of the flap 2 days before surgery and immediately after the flap elevation intraperitoneal administration POD 1–10,	Lee et al. [34]
Calcitriol	An active form of vitamin D. Upregulation of VEGF and enhancement of endothelial cell proliferation and migration	Intraperitoneal injection	Rat McFarlane skin flaps	Daily post-operative injections for 7 days	Zhou et al. [27]
Atorvastatin	A statin medication Upregulation of VEGF, bFGF, interleukin-8, angiopoietin Ang-1, Ang-2, eNOS, and hemoxidase (HO)-1. Inhibition of endothelial cells apoptosis	Oral	Rat caudally based McFarlane dorsal skin flaps	Post-operative oral administration by feeding lavage for 7 days	Chen et al. [35]
Vinpocetine	Upregulation of VEGF	Celiac injection	Rat McFarlane flap	Daily post-operative injections for 7 days	Xiao et al. [36]

Atorvastatin, a HMG-CoA reductase inhibitor used clinically in the treatment of dyslipidemia, has also been shown to have biphasic VEGF-mediated angiogenic effects [37]. At high doses, studies have suggested atorvastatin to reduce VEGF expression in various human tissues [38]. However, murine studies have shown lower concentrations to enhance endothelial cell proliferation, migration, and differentiation through upregulation of VEGF [37]. A study by Chen et al. showed 7 days of oral atorvastatin administration after dorsal skin flap elevation in rats enhanced VEGF expression and vascular density, reducing necrosis area by 20% [35].

Finally, some angiogenic therapies possess dual angiogenic and anti-inflammatory/anti-oxidant properties, such as memantine, calcitriol, and vinpocetine. Intraperitoneal injection of memantine, an excitatory amino acid receptor antagonist, calcitriol, a metabolite of vitamin D, and vinpocetine, a derivative of the alkaloid vincamine, have all been demonstrated to promote flap vascularization in rats through upregulation of VEGF production [2, 27, 36]. In addition, these therapeutics may also attenuate oxidative stress, mitigate IRI, and suppress inflammatory responses [2, 27, 36].

#### Antioxidants and anti-apoptotics

Even in the setting of adequate circulation, transient ischemia can still result in partial flap necrosis due to IRI. IRI can generate excess reactive oxygen free radicals and decrease anti-oxidant defenses, leading to endothelial cell swelling, vasoconstriction, and increased capillary permeability [39]. These changes result in damage to the mitochondrial wall and activate apoptotic pathways [2, 40–43]. Therefore, antioxidant, anti-inflammatory, and autophagic agents have been studied for their utility in reducing oxidative stress and preventing cell death in ischemic flaps.

By interacting with radicals to form less reactive products, anti-oxidant agents have shown promise in reducing IRI as well as increasing flap vascularization and survival in rat abdominal skin flaps (Table 4) [2, 27, 36, 44]. During IRI, lipid peroxidation generates malondialdehyde (MDA) which can crosslink proteins and DNA to damage cells [2, 45]. Superoxide dismutase (SOD) is a metalloprotein that can scavenge superoxide radicals to reduce oxidative stress and is one of the body’s best defenses against free radicals [2]. Angiogenic agents such as memantine, calcitriol, vinpocetine (discussed above in *“Pro-angiogenic Therapies”* section) have been shown to reduce flap tissue damage and oxidative stress in skin flaps by downregulating MDA and glutathione while increasing SOD levels.

**Table 4:** Anti-oxidant and anti-apoptotic Therapies

Pharmacologic therapy	MoA	RoA	Animal model	Treatment protocol	References
Propylthiouracil (PTU)/methimazole (MMI)	Drug-induced hypothyroidism to decrease oxidative stress, necrosis and apoptosis, enhanced NO vasodilation effect	Oral adminstration or local injection	Rat bi-pedicled dorsal random-pattern skin flap	Pre-operative oral administration for 4 weeks or immediate pre-operative injection at points 0.5, 1.5, and 2.5 cm from the cranial margin at the level of the subfascial plane of the panniculus carnosus	Rahimpour et al. [46]
Chlorogenic Acid	Removal of radicals, downregulation of MDA levels. Upregulation of SOD, reduced glutathione and superoxide dismutase levels	Intraperitoneal injection	Rat abdominal skin flap, modified McFarlane dorsal flap	Single immediate post-operative injection	Bagdas et al. [44]
Allopurinol	Inhibition of xanthine oxidase	Oral	Dogs island adipofascial cutaneous flap	Pre-operative administration for 1 week and until 48 h post-operatively	Ardakani et al. [47]
Glutathione and vitamins A, C, and E	Natural anti-oxidants, reduce free radicals	Oral or intravenous injection	Rat abdominal wall skin flap	Beta-Carotene-Vit A (Oral) Alpha-*D*-Tocopherol-Vit E (Oral) Ascorbic acid-Vit C (IV) Glutathione GSH (IV) IV injections administered 1 h post-operatively Daily oral administration for 3 days pre-operatively and a fourth dose 1–2 h post-operatively	Hayden el al. [48]

Similarly, enhanced activity of natural cellular defense mechanisms against free radicals through topical/oral administration of tocopherols (vitamin E) and retinoids (vitamin A) or injection of ascorbate (vitamin C) and glutathione have been shown to improve flap survival significantly in rats [49]. Interestingly, various studies have also demonstrated an association between hyperthyroidism and oxidant-mediated tissue damage [50, 51]. In a study by Rahimpour et al. use of anti-thyroid medications propylthiouracil and methimazole were both found to significantly improve random-pattern dorsal skin flap survival in rats by promoting cellular antioxidant activity [46].

Aside from lipid peroxidation, the xanthine oxidase (XO) system in endothelial cells is another major source of free radicals. A study by Rasti-Ardakani et al. showed that treatment pre- and post-skin flap elevation in dogs with an XO inhibitor such as allopurinol allowed flaps to tolerate a longer period of ischemia, with reduced inflammation and smaller areas of necrosis [47]. Importantly, the efficacy of allopurinol to enhance viability may be dependent on tissue- and species-specific XO activity, as this intervention was found to be less successful in preserving skin flap survival in pigs [47, 52].

#### Upregulators of autophagy

Autophagy is a highly conserved cellular degradation process to protect against metabolic stress, cellular damage, and programmed cell death [53]. Upregulation of autophagy can reduce oxidative stress‐mediated damage, enhance angiogenesis in endothelial cells Ak strain transforming (Akt) pathway activation, and thus improve the survival rate of skin flaps (Table 5) [54].

**Table 5:** Autophagy-modulating therapies

Pharmacologic therapy	MoA	RoA	Animal model	Treatment protocol	References
Calcitriol	Activation of vitamin D receptor (VDR) and autophagy-related genes	Intraperitoneal injection	Rat random pattern skin flap	Daily post-operative injection for 7 days	Chen et al. [53]
Gastrodin	Stimulation of the Nrf2/HO1 cascade, suppression of nuclear factor-κB cascade and inflammatory mediators IL-6α and IL-1β	Intraperitoneal injection	Rat random pattern skin flap	Daily post-operative injection for 7 days	Chen et al. [55]
Nobiletin	Activation of AMPK, anti-adipogenic effects, reduction of GPDH, PPARγ) and C/EBPα activity	Intraperitoneal injection	Rat random pattern skin flap	Daily post-operative injection for 7 days	Jiang et al. [56]
Andrographolide	Downregulation of Bax, CYC, and CASP3, stimulation of the PI3K/Akt-eNOS, PI3K/Akt pathway and levels of VEGF, Cadherin5, MMP9, HO1, eNOS, and SOD1	Intraperitoneal injection	Mice random pattern skin flap	Daily post-operative injection for 7 days	Jiang et al. [57]
Catalpol	Activation of SIRT1, a NAD + -dependent class III histone deacetylase	Intraperitoneal injection	Rat McFarlane flap	Daily post-operative injection for 7 days	Jiang et al. [58]
Baicalin	AMPK-regulated TFEB nuclear transcription	Oral	Rat random pattern skin flap	Daily oral administration (dissolved in DMSO and further diluted in saline) for 7 days	Zhang et al. [59]
Exenatide	Regulation of AMPK–SKP2–CARM1 and AMPK–mTOR signaling	Subcutaneous injection	Mice random pattern skin flap	Daily post-operative injection for 7 days	Li et al. [60]
Trehalose	Regulation of AMPK-regulated TFEB nuclear transcription	Intraperitoneal injection	Mice random pattern skin flap	Administration starting 12 days before operation and continued until the animals were euthanized	Wu et al. [61]
Metformin	Activation of autophagy by AMPk-mTOR-TFEB signaling pathway	Intraperitoneal injection	Rat caudally-based dorsal random pattern flap	Pre-operative and post-operative injection once daily for 7 days	Wu et al. [62]

As discussed earlier, calcitriol has been shown to upregulate VEGF production and attenuate oxidative stress, and this active form of vitamin D has been one of the best studied medications for stimulation of autophagy and relief of IRI. Intraperitoneal injection of calcitriol for 7 days postoperatively was effective in promoting autophagy-mediated angiogenesis and reducing oxidative injury in rat dorsal skin flaps, with 67.6% area of tissue survival compared to 46.8% in control animals [53]. In other rat studies, gastrodin, a chemical compound derived from the orchid *Gastrodia elata*, has also been shown to upregulate autophagy, resulting in enhanced angiogenesis and reduced cellular apoptosis [56, 55]. Similar injection for 7 days following flap elevation improved survival and increased expression of both VEGF and multiple antioxidant markers including SOD, endothelial NOS, and heme oxygenase-1 [55].

Other compounds capable of stimulating autophagy have also been shown to be effective at promoting skin flap survival in various mouse and rat studies. These include catapol, a biologically active compound found in the flowering plant *Rehmannia glutinosa*, andorgrapholide, a diterpenoid isolated from the stem and leaves of *Andrographis paniculata*, and nobiletin, a flavonoid found in citrus fruits [56, 58–57]. Each of these have been shown to positively regulate autophagy, through sirtuin 1, phophosoinositide 3-kinase/Akt, and 5′ adenosine monophosphate-activated protein kinase (AMPK) activation, respectively. Murine flap studies have shown these agents to decrease levels of pro-apoptotic markers, and promote angiogenesis, resulting in improved tissue survival [57]. Collectively, these studies highlight the protective role autophagy may play in promoting tissue survival and spotlight the promise several plant-derived compounds may possess in enhancing random skin flap survival outcomes.

#### Anti-inflammatory agents

With tissue ischemia, activation of several inflammatory signals occurs [41] which can potentiate evolution of coagulative necrosis and inflammatory cell infiltration [64]. As the extent of necrosis increases, so too does the intensity of inflammation [64]. Agents limiting the inflammatory process may thus be useful to lessen tissue damage and improve flap viability (Table 6).

**Table 6:** Anti-inflammatory therapies

Pharmacologic therapy	MoA	RoA	Animal model	Treatment protocol	References
Lidocaine	Inhibition of sodium channels, Inhibition of platelet aggregation Suppression of neutrophil mitochondrial functions Enhancement of cutaneous blood flow	Subcutaneous injection	Rat caudally-based dorsal random pattern flap	Post-operative injection once daily for 7 days	Cao et al. [65]
Apelin-13 Thermosensitiv Hydrogel	Anti-inflammatory, antioxidant, pro-angiogenic, and vasodilatory activities	Intradermal injection of hydrogel solution	Rat caudally-based dorsal random pattern flap	Single immediate post-operative injection	Zheng et al. [64]
Prussian Blue Nanozyme	Anti-inflammatory, antioxidant, anti-apoptotic, and anti-necroptotic activities	Intradermal injection	Rat chest axial-pattern flap	Pre-treatment 2 h prior to procedure	Hou et al. [43]
Topiramate	Anti-inflammatory, antioxidant, and pro-angiogenic activities	Intraperitoneal injection	Rat caudally-based dorsal random pattern flap (McFarlane flap)	Pre-operative injection 1 h and post-operatively once daily for 7 days	Ahmadzadeh et al. [66]
Colchicine	Enhancement of glutamate and NMDA receptor, inhibition of microtubule	Intraperitoneal injection	Rat cranially-based dorsal random pattern flap	Pre-operative injection 30 min	Tabary et al. [67]
Sodium valproate	Stimulation of GABA receptor stimulation and inhibition of HDAC signaling	Intraperitoneal injection	Rat cranially-based dorsal random pattern flap	Pre-operative injection 1 h prior or post-operative injection once daily for 14 days	Ala et al. [68]
Ethyl pyruvate	Anti-inflammatory, antioxidant, anti-apoptotic, antithrombotic activities	Intraperitoneal injection	Rat epigastric island flap	Post-operative injection 30 min once daily for 7 days	Kayiran et al. [42]
Heme oxygenase-1	Anti-inflammatory, antioxidant, pro-angiogenic, anti-apoptotic, and vasodilatory activities	Intraperitoneal injection	Rat left hindlimb osteomyocutaneous flap	Pre-operative injection 30 min	Zheng et al. [40]
Baclofen and bicuculline	Enhancement of GABAA a1 subunit and GABAB R1 receptor	Intraperitoneal injection	Rat cranially-based dorsal random pattern flap	Pre-operative injection 30 min	Tabary et al. [69]

Interestingly, many members of the most well-known class of anti-inflammatory medications, non-steroidal anti-inflammatory drugs (NSAIDs), have been shown repeatedly to either have unequivocal or negative effects in the setting of skin flaps [70–72]. Indeed, NSAID treatment following skin flap elevation has been associated with poor wound healing, increased incidence of infections and other complications, as well as decreased neovascularization [71]. Despite this finding, anti-inflammatory effects of a number of other therapies have been cited in their success at achieving increased skin flap viability.

Gamma-aminobutyric acid (GABA) receptors, expressed in immune cells, play a role in regulating cytokine secretion and immune cell migration [68]. Elevated GABA levels, primarily synthesized from glutamate, have also demonstrated cytoprotective capabilities, likely due to GABA's inhibitory role as a neurotransmitter [69]. Several GABA-modulating medications have been investigated as potential treatments for enhancing skin flap viability by reducing inflammation. Ivermectin (IVM), originally used as an antiparasitic agent, demonstrated enhanced flap survival in animal studies by upregulating the expression of GABA a1 subunit and GABAB R1 receptor in immune cells [69]. Sodium valproate (SV), commonly prescribed as an anticonvulsant medication, also exerts its effects through the GABA pathway, and SV administration led to increased GABA receptors and inhibition of histone deacetylase (HDAC) signaling. The application of IVM and SV both resulted in suppressed proinflammatory cytokine secretion, significantly reduced necrosis areas, and improved flap viability, indicating the potential clinical value of these agents [68, 69].

Colchicine, a decades-old anti-inflammatory drug used to treat gout, has similarly shown promise in mitigating inflammation and IRI-mediated necrosis through the glutamate pathway, ultimately resulting in enhanced skin flap survival [67]. The application of colchicine to ischemia/reperfusion injured rats reduced proinflammatory cytokines IL-6 and TNF-α and mildly increased glutamate and *N*-methyl-*D*-aspartate subunit 2A receptor expression, a glutamate receptor found on nerve cells and keratinocytes of the skin with known cytoprotective capabilities [67]. Furthermore, colchicine inhibited microtubule polymerization, which affects neutrophil adhesion, mobilization, and recruitment. Inflammation was attenuated through increased induction of the M2 macrophage phenotype, which is known for dampening of the immune response [67].

### Cell-based therapies

**Fig. 2:**
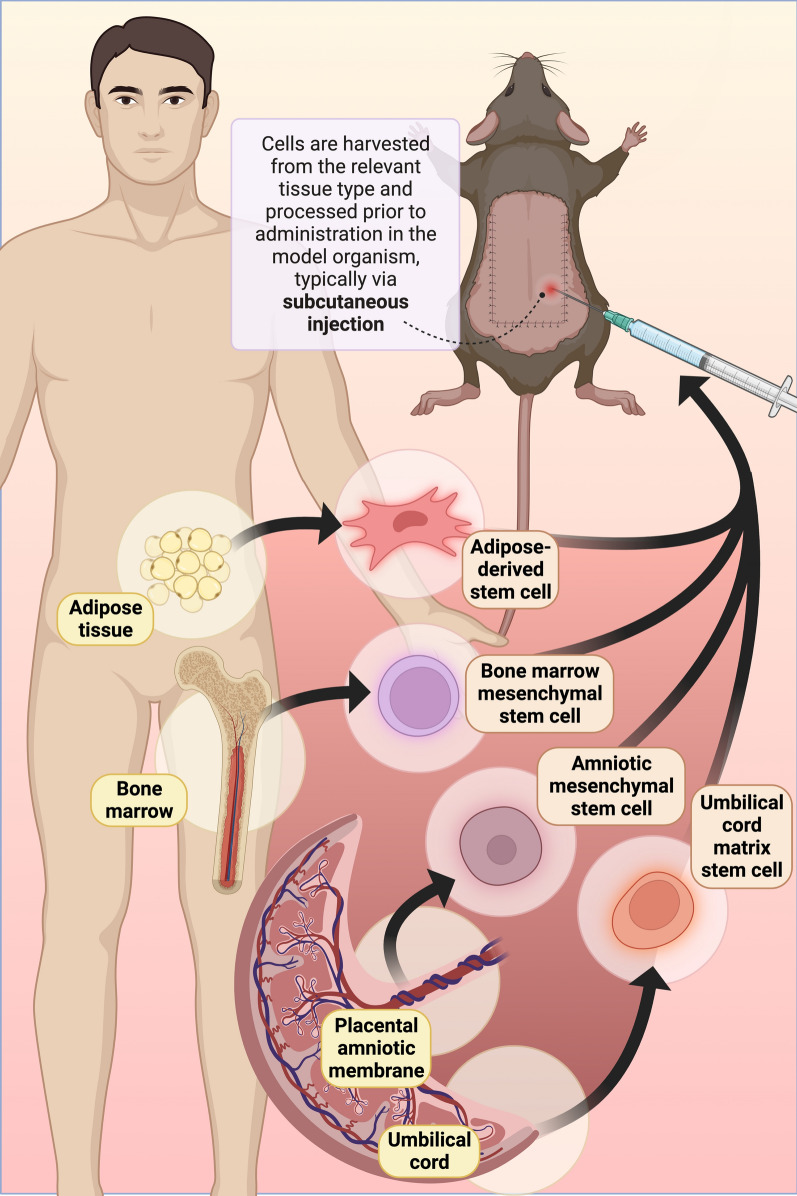
Cell-based therapies require the harvest of cells from relevant tissue type and processing prior to usage as a therapy for skin flaps. The most common sources of these cells are adipose tissue, bone marrow, placental amniotic membrane, and the umbilical cord. These cells are typically applied to preclinical murine models via subcutaneous injection, as depicted in a McFarlane flap

Cell-based therapies harness the self-renewing and regenerative capabilities of living stem cells to improve viability of skin flaps (Table 7). Cell-based therapies are a relatively new strategy to improve flap viability compared to pharmaceutical drugs and may act through various mechanisms, including direct tissue repair, immune modulation, and release of growth factors and cytokines (Fig. 2). They can potentially provide a longer therapeutic effect window due to the self-renewing and differentiating capability of the cells. Additionally, mesenchymal stem cells (MSCs) can differentiate into endothelial cells to form new vessels and significantly lower necrosis rates in rat dorsal skin flaps in ischemia–reperfusion conditions, as shown by Foroglou et al. [76].

**Table 7:** Cell-based therapies

Cellular therapy	Retrieval mechanism	Proposed benefit	Potential challenges	RoA	Animal model	Treatment protocol	References
Human subcutaneous fat extract	1. Vacuum-assisted liposuction of human adipose tissue from the abdomen, thighs, or upper arms 2. Cells are then removed from the aspirates through centrifuge	Cell-free, easy-to-prepare, lower risk of immunogenic rejection and genetic instability, and growth-factor–enriched liquid	Limited efficacy compared to cell-based therapies	Subcutaneous injection	Rat random pattern skin flap	Post-operative injections at 1.5-cm intervals of the skin flap caudally to cranially (a total of 5 injection points)	Cai et al. [29]
Adipose-derived stem cell (ADSC) therapy	1. Surgical removal of adipose tissue from the inguinal, finely minced and digested with lagenase 2. Two rounds of centrifugation to collect the layer with stromal cells, followed by flow cytometry cells sorting, induction of differentiation towards adipose cells by adipogenic substances	Abundant reserves with higher proliferating ability, easy harvest, and low donor site morbidity	Usually requires a long period of in-vitro expansion to produce a sufficient number of cells needed for transplantation	Intradermal injection	Rat random pattern skin flap	Single post-operative injection into the middle dermis along the long axis of the skin flap	Foroglou et al. [76]
Bone marrow-derived mononuclear cell	Isolation from bilateral femurs and tibias. Cells were then isolated using a strainer mesh, centrifugation, followed by a Ficoll-paque density gradient separation	Direct transplantation without in vitro cell expansion, enhances angiogenic growth factors bGFG and VEGF	Limited source	Subcutaneous injection	Rat random pattern skin flap	Injected at 10 points along the axis of the flap from the base to the distant end 2 days pre-operatively	Yang et al. [73]
Human amniotic membrane (h-AM)	AMs were obtained from placentas at the time of elective cesarean sections from overall healthy donors Then, they were manually separated from the chorion, placed on the nitrocellulose membrane and cut into pieces small sheets MAMs: The AM sheets are homogenized into microparticles with macro homogenization, freeze dried and filtered through a metal mesh o obtain microparticles	Cellular components with high tensile strength and tissue modeling power		Transplantation of the amniotic membrane sheet (AMS) Smearing of micronized amniotic membrane (MAM)	Rat random pattern skin flap	AM sheet was transplanted into the flap site MAM was smeared into the wound surface	Nazanin et al. [74]
Human umbilical cord matrix stem cells	Human umbilical cords were obtained from a local obstetrician from full-term Caesarian section births. Umbilical arteries and veins were removed. Whole-cell lysates were made from Wharton’s jelly cells by standard techniques using a lysis buffer	Robust proliferation and differentiation power for harvest in large quantity, High plasticity, and low immunogenicity		Subcutaneous injection	Mice axial epigastric skin flap pattern	Single post-operative injections at 10 evenly distributed points along the axis of the base of the flap to the distant end, each 1 cm apart	Leng et al. [28]
Mesenchymal stem cells	BM was flushed from the bones, isolated, and screened for MSC markers to separate BMMSCs. BMMSCs were then cultured, separated, and centrifuged	Most commonly used stem cell source due to its high efficacy to flap viability	Limited source and invasive harvesting procedures	Subcutaneous injection	Rat random pattern skin flap	Single post-operative injections of the BMMSCs at 12 points on each flap	Chehelcheraghi et al. [75]

MSCs, and particularly adipose-derived stem cells (ADSCs), are the most frequently studied cell types [3], and have been associated with anti-oxidant, vasodilatory, anti-inflammatory, and angiogenic effects [76]. Several sources of MSCs, including ADSCs from lipoaspirate, bone marrow, and human umbilical cord matrix stem cells (hUCMs), have been studied and demonstrate different benefits and drawbacks in terms of therapeutic potential and ease of harvest/isolation.

ADSCs have the benefits of abundant reserves with high proliferating ability, simple harvest with liposuction, and low donor site morbidity. However, the harvested lipoaspirate is initially impure and requires either a large volume or a long period of in vitro expansion to produce a sufficient number of ADSCs for transplantation [73]. ADSCs have been found to effectively reduce distal skin flap necrosis, with a meta-analysis finding that treatment resulted in an absolute risk reduction in necrotic skin area of 22.37% [77]. Augmentation of ADSCs with exosomes stimulated by hydrogen peroxide [78] and preconditioning of ADSCs with hypoxia [79] have been shown to amplify these therapeutic effects. Several studies have isolated various components of ADSCs, such as extracellular vesicles and exosomes and have shown these to independently improve skin flap survival, suggesting contribution of these components to the therapeutic value of these cells [80–83].

MSCs can be isolated from bone marrow and used in cell-based therapies to improve flap viability. Interestingly, while these cells have been shown to stimulate VEGF activity, neovascularization, and collagen density in rat random-pattern skin flaps, they do not appear to have a beneficial effect on the fibroblast number or other biomechanical parameters in flap wound healing [75]. The limited supply and invasive harvesting procedures necessitated by stem cell therapies from the bone marrow are an important limitation their clinical tranlatability [28].

Human umbilical cord matrix stem cells (hUCMs) are derived from human umbilical cord Wharton’s jelly and are more easily isolated in a large number. Compared to BM-MSCs, hUCsM may have more robust proliferation and differentiation capabilities, greater plasticity, and lower immunogenicity. A study by Leng et al. showed that hUCMs promote vascularization by increasing capillary density, enhancing angiogenic growth factors such as VEGF and bFGF levels, and improving the survival of ischemic epigastric mouse flap models [28].

Tissue engineering approaches have also been applied to the field of cell-based therapies for skin flap survival, with a 2022 study by Nazanin et al. exploring the use of placental amniotic membrane as a scaffold source to improve flap viability. From the amniotic membrane, researchers have studied the utility of amniotic membrane sheet (AMS) and micronized amniotic membrane (MAM) products, which contain human amniotic MSCs, a number of angiogenic growth factors, and ECM components to impart tensile strength [74]. While both products were found to be effective treatments to improve rat flap survival, each imparted different therapeutic benefits [74]. Specifically, transplantation of MAM improved the organization of collagen tissue and angiogenesis rate, while AMS had more profound anti-inflammatory effects [74]. AMS treatment was also found to increase epithelialization of keratinocytes and the in-growth of fibroblasts during wound healing [74].

In summary, cell-based therapies offer a promising avenue for enhancing skin flap viability through the utilization of various stem cell types, including mesenchymal stem cells (MSCs), adipose-derived stem cells (ADSCs), and human umbilical cord matrix stem cells (hUCMs). Additionally, the incorporation of tissue engineering approaches, such as placental amniotic membrane, adds further depth to this innovative field of research and presents diverse avenues for improving the survival and quality of skin flaps. While evidence suggests promising utility for stem cells in improving skin flap viability, many factors produced by these cells and their specific effects remain poorly understood. Further characterization of the factors produced by distinct cell lines and their impact on the post-operative skin flap environment will provide a more granular understanding of the possible clinical utility of stem cells for improvement of skin flap viability.

### Translatability challenges and future directions

Preclinical investigation of pharmacologic and cell-based therapies has attempted to improve viability through a variety of pathways, drug classes, and stem-cell types. Despite promising results, the translation of these therapies to common clinical practice has yet to be seen. The reasons for this are complex, though several include negative drug side effects, complicated treatment regimens, and high economic/logistic costs. Calcium channel blockers like Nifedipine, for example, can cause palpitations, edema, and constipation, while the antiepileptic Sodium valproate can impede hair growth and amplify weight gain when administered systemically. On the topic of administration, treatment protocols may often be demanding, with some drugs requiring daily injections due to short half-lives and low plasma concentrations. The cost of these agents can also be prohibitive for practical translatability, particularly for growth factors like VEGF and cell-based therapies. Cell-based options are accompanied by several logistical barriers which contribute to their high costs, such as sourcing, expansion, and delivery. Clinical limitations such as immuno-rejection and genetic stability exist as well.

The future of clinical practices to enhance skin flap viability requires clear understanding of pro-survival pathways, as well as validation of results in more rigorous studies. Many of the experiments conducted in this field have utilized small animal models, and large animal models as well as clinical trials with long-term follow-up will ultimately be needed [77].

Furthermore, with promising results demonstrated by induction of ischemia [84] to promote angiogenesis prior to creating a skin flap, interest in preconditioning treatments to prepare a skin region for use as a skin flap has emerged. Studies have reported promising results with hyperbaric oxygen therapy [85, 86] and local warming of the skin using a heat blanket prior to surgery, which have been shown to enhance skin flap survival in pigs [87]. Additionally, advances in flap care are moving toward the direction of combination therapies that promote viability by addressing a variety of pathways simultaneously [22, 88, 89]. For example, one study simultaneously used hyperbaric oxygen therapy to promote angiogenesis and nitroglycerin to promote vasodilation [90]. Another study investigated the synergistic action of azelaic acid, minoxidil, and caffeine to protect against IRI by targeting parallel anti-apoptotic, anti-inflammatory, and antioxidant pathways [22].

As techniques advance, so, too has drug delivery technology. Novel delivery mechanisms for pharmaceutical therapies such as an injectable thermosensitive hydrogel [64] and photocrosslinked gelatin hydrogel implants [91] have been introduced for optimized drug delivery. These gel-based biomaterials allow for controlled release platforms that can be applied directly to the wound bed prior to flap closure, topically to the flap site, injected directly into the flap, or incorporated into dressings [64]. Nanoparticles that encapsulate drugs and target specific tissues or cells within a flap offer controlled release and drug protection, ultimately enhancing delivery and reducing systemic side effects [92].

Since survival rate of compromised skin flaps correlate inversely with time, treatment following the first signs of necrosis—particularly within the first 72-h window—is important to provide timely interventions to minimize the risk of flap necrosis [93, 94]. A recent paper reported bioengineering sensors that use non-invasive electrical measurements to monitor biochemical parameters, such as pH value or dissolved oxygen concentration, and biophysical parameters, like blood flow and temperature, to relay real-time information regarding flap viability may thus be of future value [92]. These sensors allow for timely detection of signs of flap failure, allowing an opportunity for intervention and, ultimately, improved survival [95].

## Conclusion

Translational work in the field of skin flaps aimed at augmenting viability has demonstrated significant potential through diverse approaches, drug classes, and cell types. Despite promising results, the translation of these therapies to common clinical practice remains elusive. Challenges such as clinical side effects, complex treatment protocols, high economic and logistic costs, dosing regimens, and incomplete understanding of underlying molecular pathways have hindered their widespread adoption. To overcome these obstacles and pave the way for successful clinical implementation, future research must focus on elucidating the intricate mechanisms of action, conducting rigorous and comprehensive preclinical and clinical trials, adopting a holistic approach through combination therapies, exploring novel delivery methods, and leveraging technological advancements for flap monitoring. Moreover, validating results in large animal models and conducting long-term clinical trials will be crucial for establishing the efficacy and safety of these interventions. Recent advances in preconditioning treatments, combination therapies, drug delivery systems, and monitoring technologies offer hope for overcoming the existing challenges and ultimately enhancing skin flap viability in clinical settings. As the safety and efficacy of pharmaceutical agents in flap viability continue to progress, they hold the potential to significantly improve patient outcomes and contribute to advancements in reconstructive surgery.

## Data Availability

Not applicable**.**
